# Digital exercise interventions for improving measures of central obesity: a systematic review

**DOI:** 10.1007/s00038-020-01385-4

**Published:** 2020-05-14

**Authors:** Marcel Ballin, Andreas Hult, Sabine Björk, John Dinsmore, Peter Nordström, Anna Nordström

**Affiliations:** 1 Department of Community Medicine and Rehabilitation, Unit of Geriatric Medicine, Umeå University, Umeå, Sweden; 2 Department of Public Health and Clinical Medicine, Section of Sustainable Health, Umeå University, Umeå, Sweden; 3 Department of Community Medicine and Rehabilitation, Section of Sports Medicine, Umeå University, Umeå, Sweden; 4 Department of Nursing, Umeå University, Umeå, Sweden; 5 Trinity Centre for Practice and Healthcare Innovation, School of Nursing and Midwifery, Trinity College Dublin, Dublin, Ireland; 6 School of Sport Sciences, UiT The Arctic University of Norway, Tromsø, Norway

**Keywords:** Digital health, Physical activity, Visceral adipose tissue, Obesity

## Abstract

**Objectives:**

We aimed to systematically review the potential benefits of digital exercise interventions for improving measures of central obesity including visceral adipose tissue (VAT) and anthropometric surrogates for VAT in overweight or centrally obese adults aged 18 or over.

**Methods:**

A systematic literature search was conducted in three databases up until March 2020 (PROSPERO registration nr CRD42019126764).

**Results:**

*N* = 5 studies including 438 participants (age 48–80) with body mass index ≥ 25 kg/m^2^ met the eligibility criteria and were included. The duration of the interventions ranged from 8 to 24 weeks. No study measured the primary outcome VAT, although in *N* = 4 studies, waist circumference (WC) decreased by between 1.3 and 5.6 cm in the intervention groups.

**Conclusions:**

This systematic review shows that there is no evidence for the effects of digital exercise on VAT, although digital exercise may decrease WC. These findings highlight the need for additional randomized controlled trials to confirm the findings with respect to WC, and to further investigate the effects of digital exercise on VAT. Together, this may have important implications for reducing the burden of physical inactivity and obesity.

## Introduction

Obesity and physical inactivity remain among the leading causes of mortality and major risk factors for cardiovascular disease (CVD) (Bowman et al. 2017; Forouzanfar et al. 2016; Kopelman 2000; Lee et al. 2012; World Health Organization 2009; Yusuf et al. 2004), despite their modifiable nature making them prone to interventions. Specifically, central obesity which is characterized by an excessive deposition of visceral adipose tissue (VAT) in the abdominal cavity has been more strongly associated with CVD and mortality than general obesity (Sahakyan et al. 2015; Sharma et al. 2016; Yusuf et al. 2005). It has also been shown that improving modifiable risk factors for CVD may also reduce further comorbidities later in life including disability and frailty (Atkins et al. 2019).

Despite the vast amount of research showing that supervised exercise, regardless of caloric restriction and weight loss, has positive effects on central obesity (Kay and Fiatarone Singh 2006; Verheggen et al. 2016; Vissers et al. 2013; Wewege et al. 2017), the prevalence of obesity and physical inactivity remains high (Guthold et al. 2018; World Health Organization 2018). Therefore, there is a need to explore novel approaches for promoting physical activity (PA) to counteract the detrimental effects of insufficient activity and obesity and their economic burden (Ding et al. 2016; Oldridge 2008; Withrow and Alter 2011). Over the past two decades, there has been a dramatic increase in the Internet users (Internet Society 2017) and subsequently a rise also in the number of digital health interventions. The previous reviews have concluded that digital health interventions may have positive effects on outcomes such as PA and quality of life (Cotie et al. 2018; Foster et al. 2013; Geraedts et al. 2013; Jahangiry et al. 2017; Schäfer et al. 2018), and among the unique advantages of these interventions are cost-efficiency, accessibility and convenience, including 24-h access to intervention material (Joseph et al. 2014; Lewis et al. 2010; Oh et al. 2005; World Health Organization 2016). In light of this, digital tools (e.g., web-based or smart device applications) may be plausible for delivering wide-spread, effective and cost-efficient exercise interventions in a home-based setting.

However, the previous research has not produced consistent results in terms of the effects of digital health interventions on anthropometric measures of central obesity, reporting both significant and non-significant findings (Cotie et al. 2018; Seo and Niu 2015). In addition, these reviews included multicomponent interventions and did not restrict their inclusion criteria to exercise-only interventions, thus making it impossible to determine the exercise-specific effects on measures of central obesity. Therefore, the aim of the present study was to systematically review the potential benefits of digital exercise-only interventions for improving measures of central obesity in overweight or centrally obese adults. Outcome measures included VAT, waist circumference (WC), waist–hip ratio (WHR), sagittal abdominal diameter (SAD), body fat percentage (BFP), body weight (BW) and body mass index (BMI).

## Methods

The methods and protocol of the present systematic review were prospectively registered with PROSPERO (registration number: CRD42019126764), and PICOS (Population, Intervention, Comparison, Outcome, Study design) was applied to define the research question. Reporting of the present systematic review was based on the PRISMA guidelines (Moher et al. 2009).

### Eligibility and exclusion criteria

#### Population

For the present systematic review, studies were included if the sample of participants was 18 years of age or older and considered predominantly overweight (BMI > 25 kg/m^2^) or centrally obese (WC > 88 cm for women and > 102 cm for men) (World Health Organization 2000). Trials with either non-ambulatory or non-community dwelling participants or participants not meeting the above criteria for overweight or central obesity were excluded from this review.

#### Intervention

Digital exercise-only interventions were included. Exercise was defined as planned, structured and purposive physical activities with the objective of maintaining or improving physical fitness (Caspersen et al. 1985). The term digital exercise intervention was defined as an intervention where exercise was assigned to and accessible for the participants using digital tools, e.g., usage of websites, smartphone applications, video or audio instructions, messaging services or videogames (World Health Organization 2016).

#### Comparisons

Outcome data were extracted for the intervention group (IG) in each study, and for the control group (CG) when one was present, as long as the CG had not received an exercise intervention.

#### Outcomes

The primary outcome was VAT (area, volume or grams). Additional outcomes were WC (cm), WHR (cm), SAD (cm), BFP (percentage), BW (kg) and BMI (kg/m^2^). Studies were required to report data on at least one of the central obesity measures (VAT, WC, WHR) to be included.

#### Study design

Studies were required to be prospective intervention studies in terms of either randomized controlled trials, quasi-experimental studies or single-arm intervention studies.

### Search strategy

A systematic search strategy was developed during November–December 2018 using search terms related to central obesity, exercise and digital health (“Appendix”). This work was conducted by two librarians and in collaboration with the authors. The initial literature search was performed in January 2019. A supplementary search was performed in March 2019, followed by another one in March 2020. Searches were performed in PubMed, CINAHL and SPORTDiscus. Finally, the authors supplemented the electronic searches by screening the reference lists of already included studies.

### Study selection

The results from the electronic searches were extracted into a database where M.B performed the screening of titles and subsequently abstracts to identify eligible articles and remove irrelevant articles as well as duplicates. The remaining full-text articles were then reviewed independently by M.B, A.H and S.B to ensure that they met the inclusion criteria. The results from the independent review process were then compared, and any discrepancies were discussed until consensus was reached.

### Data extraction

Data that were extracted in the present review included the following: authors and publication year; participant characteristics in terms of age, sex and population; sample size; study design; intervention duration; intervention specifics; intervention adherence; control group specifics; outcome measures including VAT, WC, WHR, SAD, BFP, BW, BMI (baseline, post intervention, mean change, standard deviations, standard errors, *P* values). Extracted data were presented descriptively and no analyses were performed.

### Risk of bias in individual studies

The risk of bias in the studies included in the review was rated independently by M.B, A.H and S.B, following the same consensus procedure employed for study selection. The Cochrane Collaboration Tool (Higgins et al. 2011) was used for assessing risk of bias, where seven domains are judged as low, unclear or high risk of bias. These domains are as follows: random sequence generation; allocation concealment; blinding of participants and personnel; blinding of outcome assessment; incomplete outcome data; selective reporting; other bias. For the purpose of this review, other bias was judged depending on whether central obesity was measured using anthropometric surrogates or direct measures of using imaging techniques, considering the latter are considered the gold standard for quantifying visceral adiposity (Shuster et al. 2012). A summary figure of the assessed bias of the included studies was created using Review Manager v.5.3 (Copenhagen: The Nordic Cochrane Centre, The Cochrane Collaboration 2014).

## Results

### Study selection

**Fig. 1:**
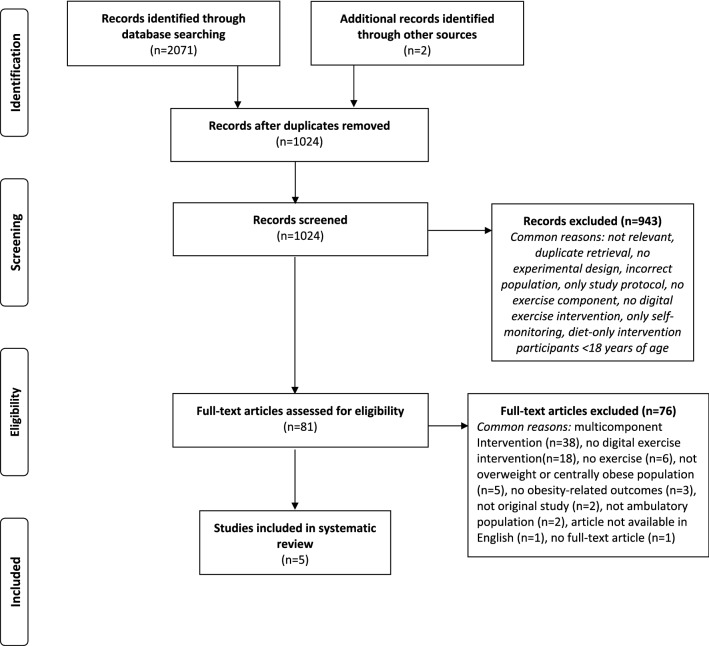
PRISMA flowchart of the literature search and study selection process

The search identified *N* = 2071 potential articles from three databases: PubMed *(N* = 1499), CINAHL *(N* = 390) and SPORTDiscus *(N* = 182) in addition to *N* = 2 articles that were manually retrieved based on identified secondary analyses (Llanos et al. 2014; Vroege et al. 2014). Following removal of duplicates (*N* = 1049) and screening of abstracts and titles, 81 full-text articles were assessed for eligibility. From these 81 articles, *N* = 5 studies met the inclusion criteria and were included in this review. The majority of excluded articles at full-text level resulted from studies which included multicomponent interventions (*N* = 38) and a lack of a digital exercise intervention (*N* = 18). A detailed flowchart of the study selection process and reasons for exclusion are provided in Fig. 1.

### Study characteristics

Participant and study characteristics of each of the five included studies are presented in Table 1. The total number of included participants was 438. Across the five studies, the mean age of the study populations ranged from 48 to 80 years, and the mean BMI ranged from 25.0 to 32.5 kg/m^2^. The gender distribution of participants ranged from 11% to 100% female participants. All studies were published between 2010 and 2018, and the interventions lasted between 8 and 24 weeks.

**Table 1:** Population and study characteristics of the five studies included in the systematic review

Study	Population	Total sample size (IG + CG)	BMI mean (SD)	Age mean (SD)	Sex (%F)	Study design	Intervention and control group specifics	Outcomes
Akinci et al. (2018)	Patients with type 2 diabetes	33 (22 + 21)	I: 32.5 (4.4) C: 32.2 (5.1)	I: 50.2 (6.5) C: 53.6 (6.7)	87.9%	8-week RCT	The IG were provided with an Internet-based exercise program consisting of both aerobic and resistance exercises. The CG were given a brochure containing information on the benefits of physical activity and exercise	WC, BMI
David et al. (2012)	Postmenopausal women	71 (35 + 36)	I: 31.0 (3.6) C: 32.0 (4.4)	I: 57 (5) C: 57 (5)	100%	12-week randomized feasibility trial	Both groups received a walking intervention using pedometers and an Interactive Voice System (IVR). Step goals were set based on baseline physical activity levels. The daily step goals were progressively increased until participants reached 10,000 steps/day. Participants received daily messages through their phones, in addition to answering automated calls from the IVR systems. The only difference between the groups was that the IG had the possibility to call a human coach through the IVR system. Thus, the effects on the outcomes were only reported for all participants as one group	WC, WHR, BMI, weight
Pressler et al. (2010)	Overweight and sedentary employees	50	I: 28.6 (1.9) C: 28.8 (2.5)	48 (25–60)^a^	11%	12-week RCT	Participants received a structured Internet-delivered exercise program. The program included an interactive web-calendar where participants were able to choose from a variety of different activities including endurance and strength workouts. Weekly goals were established with the aim of increasing activity up to 1500 MET*min/week. Exercise intensity was prescribed based on the baseline assessment. The exercise sessions were planned and provided as individual workouts	WC, BMI, BFP
Thompson et al. (2014)	Sedentary and overweight older adults	49 (25 + 24)	25.0–40.0^b^	I: 79.1 (8.0 C: 79.8 (6.0)	81%	24-week randomized cross-over trial	The IG received “Go4Life” educational material and counseling including education on different exercise modalities, goal-setting and individual plan-building. They IG received an accelerometer with subsequent feedback based on activity levels. The CG did not receive “Go4Life” and only received an accelerometer without feedback	WC, BFP, weight
Wijsman et al. (2013)	Independent older adults	235 (119 + 116)	I: 28.9 (4.7) C: 29.1 (4.7)	I: 64.7 (3.0) C: 64.9 (2.8)	I: 39.5% C: 42.2%	12-week RCT	The IG received a web-based intervention including an activity monitor, personal website and personal online coach. Based on current activity levels, personal goals were established. Activity targets were increased progressively throughout the course of the intervention. The IG were living as usual	WC, WHR, BMI, BFP, weight

*BFP* body fat percentage, *BMI* body mass index, *CG* control group, *IG* intervention group, *MET* metabolic equivalent, *RCT* randomized controlled trial, *WC* waist circumference, *WHR* waist–hip ratio

^a^Indicates median and range

^b^Indicates range

### Intervention specifics

The nature of digital exercise intervention varied widely between the five included studies. Akinci et al. (2018) randomized participants to an IG and a CG. The CG received a brochure with information about health and exercise while the IG performed three exercise sessions for eight weeks, where each session lasted 50–60 min. The exercise program consisted of both endurance- and strength exercise and participants accessed the program at home using an online platform. There, they subscribed to a website where the exercise program in terms of online videos was available. In addition, they were instructed to report at the website following completion of a session, and they were also reminded not to forget to follow the exercise program.

David et al. (2012) randomized participants to an IG and a CG. Both groups were offered a 12-week walking intervention and provided pedometers as well as an Interactive Voice Response (IVR) system through mobile phones. Participants were given step goals based on their baseline level of PA. These daily targets were progressively and individually increased so that participants would reach 10,000 steps/day. Each day participants received messages through their phones, in addition to answering automated calls from the IVR systems. Within the scope of the calls, participants would answer whether they had completed their daily step goal, their self-efficacy toward completing the daily goal and how their day had been in general. The only difference between the groups was that the IG had the possibility to contact a coach through the IVR system, although these interactions were not compulsory for the participants. Thus, the authors of the study reported the effects on the outcome measures for all participants combined together, rather as separate effects in each group.

Pressler et al. (2010) originally randomized participants to an intervention IG and a CG. In the present review, only the IG was assessed as the participants in the CG received an all too similar intervention as the IG, hence, an appropriate comparison with the IG was not possible and justified. The intervention consisted of a 12-week long individually planned and structured exercise program delivered as an interactive web-calendar. Using the calendar, participants could choose from a variety of workouts. Each week, they would perform three moderate-intensity endurance workouts and one strength training session, with each session lasting between 30 and 70 min. Exercise intensity was prescribed as 60–70% of maximum heart rate and based on baseline assessments. Participants also documented their heart rate on the interactive platform. Individual weekly goals were also established.

In the study by Thompson et al. (2014), participants were randomized to an IG and a CG. The IG received the “Go4Life” material and counseling for 12 weeks, as developed by the National Institute on Aging (http://www.Go4Life.nia.nih.gov) The “Go4Life” included education on different exercise modalities, goal-setting and individual plan-building. In addition, participants were given an accelerometer which provided feedback based on their PA levels throughout the study. Participants developed their own individual plan and goals, which they discussed once every week via phone calls with a counselor. The aim was for all participants to increase their PA level by at least 20% from baseline. The CG only received an accelerometer which did not provide feedback to the participants.

Wijsman et al. (2013) randomized subjects to an IG and a wait-list CG. The intervention was a 12-week web-based PA program based on health behavior-change models, with the aim of increasing daily PA, tailored specifically for each participant depending on their current ability. The program provided a personalized goal for the participants, who also received an accelerometer, a personal website and a personal online coach. The coach and the participant were in regular contact throughout the intervention period, and the coach was regularly providing updates and exercise advice to the participant, depending on data uploaded from the accelerometer. The goals were progressively increased throughout the 12-week intervention period. The CG did not receive any specific instructions regarding PA.

### Results of individual studies

Results of the interventions on measures of central obesity are provided in Table 2. Three of the studies presented data for both an IG and a CG. In two studies, only data from the IG was presented due to: (1) data only being reported for all participants combined as one group, and (2) lack of plausible CG.

**Table 2:** Results of digital exercise interventions on measures of central obesity in the five studies included in the systematic review

Study	Outcome	Intervention group (Δ)	Control group (Δ)	*P* for difference
*Studies with a control group*				
Akinci et al. (2018)	WC (cm)	− 5.6 ± 8.8^a^	− 0.2 ± 5.7^a^	0.006
	BMI (kg/m^2^)	− 0.7 ± 3.4^a^	− 0.7 ± 3.4^a^	0.29
Thompson et al. (2014)	WC (cm)	− 1.6 ± 7.6^a^	− 2.0 ± 7.16^a^	0.85
	BFP (%)	0.01 ± 1.5^a^	− 0.3 ± 1.8^a^	0.58
	Weight (kg)	− 1.0 ± 2.3^a^	− 1.0 ± 1.9^a^	0.97
Wijsman et al. (2013)	WC (cm)	− 2.3 ± 0.4^b^	− 1.3 ± 0.3^b^	0.036
	WHR	− 0.008 ± 0.004^b^	− 0.001 ± 0.003^b^	0.16
	BMI (kg/m^2^)	− 0.5 ± 0.1^b^	− 0.3 ± 0.1^b^	0.068
	BFP (%)	− 0.6 ± 0.2^b^	0.1 ± 0.2^b^	0.025
	Weight (kg)	− 1.5 ± 0.3^b^	− 0.8 ± 0.2^b^	0.046

Study	Outcome	Intervention group, Δ	*P*
*Studies without control group*			
David et al. (2012)	WC (cm)	− 1.3 ± 0.6^b^	0.049
	WHR	− 0.01 ± 0.01^b^	0.308
	BMI (kg/m^2^)	− 0.3 ± 0.1^b^	0.045
	Weight (kg)	− 0.9 ± 0.3^b^	0.017

Study	Outcome	Intervention group, baseline	Intervention group, follow-up	*P*
Pressler et al. (2010)	WC (cm)	100.5 ± 7.9^a^	98.0 ± 7.8^a^	0.001
	BMI (kg/m^2^)	28.6 ± 1.9^a^	28.3 ± 2.0^a^	0.12
	BFP (%)	30.1 ± 5.2^a^	29.2 ± 5.7^a^	0.22

*BFP* body fat percentage, *BMI* body mass index, *WC* waist circumference, *WHR* waist–hip ratio

All data are presented as mean values with ^a^standard deviation and ^b^standard error

#### Outcomes

None of the included studies measured VAT. However, all of the studies measured WC and two of the studies also measured WHR (David et al. 2012; Wijsman et al. 2013). BMI was measured in four of the studies, while body fat percentage (BFP) and BW were measured in three studies. For the three studies where data were presented for both an IG and a CG (Akinci et al. 2018; Thompson et al. 2014; Wijsman et al. 2013), there were significant decreases in WC in favor of the intervention within two of the studies. Specifically, in the study by Akinci et al., the IG decreased WC by a more than 5 cm compared to the CG (*P *< 0.05) in the absence of effect on BMI. In the study by Wijsman et al. (2013), the IG decreased WC (− 2.3 cm vs − 1.3 cm *P *< 0.05), BFP (− 0.6% vs 0.07%, *P *< 0.05) and BW (− 1.5 kg vs − 0.8 kg, *P *< 0.05) compared to the CG. Thompson et al. (2014) did not observe significant differences between the IG and the CG on any outcome measure.

For the studies where only data from the IG were presented, David et al. (2012) found significant effects on WC, BMI and BW following the intervention (*P *< 0.05 for all, Table 2), where WC decreased by 1.3 cm and BW decreased by 0.9 kg. Pressler et al. (2010) found a 2.5 cm decrease in WC (*P* < 0.05) in their IG, but no significant effects on BMI or BFP.

### Adherence to the interventions

In the study by Akinci et al. (2018), over 52% of the participants in the IG were excluded due to insufficient adherence to the intervention. Specifically, 28.5% of the participants never visited the website and 23.8% failed to complete the exercise program for three consecutive weeks. It is somewhat unclear if results are presented only from remaining participants or from additional imputed data. Only one individual was lost to follow-up in the study by Thompson et al. (2014). Adherence was not specified, and the intervention did not result in the intended aim of increasing PA at outcome assessment. In the study by Pressler et al. (2010), there was a 24% lost to follow-up in the IG between baseline and outcome assessment. The remaining participants within the IG reported completion of 47% of the scheduled workouts and were all included in the final analysis. Wijsman et al. (2013) did not report adherence to weekly activity level targets; however, 91% of the participants completed the intervention program. In the IG, two participants were lost to follow-up and an additional three discontinued the intervention. There were no participants lost to follow-up in the CG, but four discontinued the intervention. All participants with complete outcome assessments were included in the analysis. In the study by David et al. (2012), 55% of the individuals completed the outcome assessment and were included in the analysis, with 51% of the calls made by the IVR system answered.

### Risk of bias within studies

**Fig. 2:**
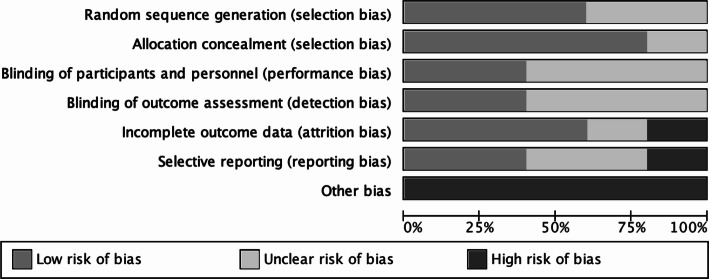
Risk of bias graph showing percent of studies in the systematic review with low, high or unclear risk of bias across domains according to The Cochrane Collaboration Tool

The summary of risk of bias across all included studies is provided in Fig. 2. The highest risk of bias resulted from ‘other bias’, in terms of the absence of direct techniques for measuring central obesity which was the case for 100% of the included studies. Similarly, 60% of the studies did not state whether the outcome assessment was blinded or not. Additionally, in one study Akinci et al. (2018), there was a high risk of attrition bias given that inappropriate methods were employed for handling missing data, resulting in potentially over-estimated effects. Also, data on changes in body fat were not reported in this study despite stated in the pre-registered study protocol. While these values would have been of benefit to the present review, unfortunately, they were not reported. This would have been of value for the present systematic review and thereby resulted in a high risk of reporting bias. Blinding of participants was either not performed or sufficiently described in 60% of the studies. In general, blinding is not possible in exercise trials due to participants usually being aware of whether they receive the intervention or not. For this reason, the studies that did not address blinding were therefore not considered as a source of high risk of bias in the present systematic review.

## Discussion

The purpose of the present study was to systematically review the existing literature and provide an overview of the potential benefits of digital exercise interventions for improving measures of central obesity. We found that the potential benefits of digital exercise for decreasing VAT have not yet been investigated. However, in four studies, WC decreased by between 1.3 and 5.6 cm in the IGs. In addition, there were a few positive, albeit inconsistent, findings on overall obesity-related outcomes including BMI, BW and body fat.

Since 1980, the prevalence of obesity has doubled in more than 70 countries (Afshin et al. 2017), and it is projected to continue to increase for the foreseeable future (Ward et al. 2019). At the same time, physical inactivity has been labeled as a pandemic (Kohl et al. 2012), and recent reports show no signs of improvements for the past 15 years (Guthold et al. 2018). With this in mind, the current rapid growth in the number of internet users (Internet Society 2017) could offer potential advantages in terms of prevention and treatment, as the World Health Organization has proposed that digital health interventions should be further developed and implemented to support individual health and health care systems (World Health Organization 2016). Previous reviews have reported inconsistent findings in terms of the effects of digital health interventions on obesity-related outcomes (Cotie et al. 2018; Seo and Niu 2015). A limitation in these reviews is that they did not formalize their criteria to include exercise-only interventions, which is essential in order to elucidate the exercise-specific effects on obesity-related outcomes. To our knowledge, this was the first systematic review to evaluate the potential benefits of digital exercise-only interventions for improving measures of central obesity. The findings highlight the importance of employing measures of obesity beyond BMI when evaluating changes in body composition following digital exercise interventions. Specifically, we observed more inconsistent findings on BMI compared to WC, which is supported by previous meta-analyses which have shown that supervised exercise interventions may effectively improve measures of central obesity in terms of reducing VAT and WC despite the absence of significant weight loss (Vissers et al. 2013; Wewege et al. 2017). While we were unable to determine the benefits of digital exercise for decreasing VAT, it is nonetheless promising that the present review suggests that digital exercise may decrease WC in the absence of a hypocaloric diet. This likely also has clinical relevance given that measures of central obesity are better indicators of health outcomes than BMI (Czernichow et al. 2011; Nordström et al. 2016), and have been associated with increased risk of CVD and mortality (Czernichow et al. 2011; Onat et al. 2004; Rexrode et al. 1998), even in normal-weight individuals (Sahakyan et al. 2015; Sharma et al. 2016). In a pooled analysis of 11 prospective studies including 650,000 adults and over 78,000 deaths, it was shown that 5 cm increments in WC increased the relative risk of mortality by 7–9% across a wide range of BMI categories (Cerhan et al. 2014). Similarly, when changes in WC were studied in relation to all-cause and CVD-mortality, 5 cm increments in WC was associated with a 51% and 84% increased relative risk of all-cause and CVD-mortality in men during a follow-up period of 16 years (Mulligan et al. 2019). For women, the relative risks for the same outcomes were 25% and 15%, respectively. Even smaller increments may also have clinical significance as demonstrated in a meta-regression analysis where as little as 1 cm increment in WC was associated with increased risk of CVD (de Koning et al. 2007). To this end, interventions that can improve measures of central obesity clearly has important implications, although when interpreting the findings from the present review it is critical to consider that merely five studies were included in this review, and none of these measured VAT directly. It would therefore be valuable if future studies aim for objective measurements of central obesity.

With regards to the inverse association between PA and the risk of CVD (Kyu et al. 2016; Lear et al. 2017; Li and Siegrist 2012; Wahid et al. 2016), the ability to remotely provide individuals with personalized digital exercise interventions may have significant public health value with respect to accessibility and cost-efficiency (Joseph et al. 2014; Lewis et al. 2010; Oh et al. 2005; World Health Organization 2016). Specifically, provision of digital health applications may be a cost-efficient way to intervene on the declining physical fitness of people living in rural communities (Ekblom-Bak et al. 2019), and thus should be of interest to health care providers and stakeholders. In this sense, it is interesting that a recent systematic review of 22 studies on older people reported a very high adherence rate, around 90%, to digital exercise interventions, although the interventions included some level of supervision (Valenzuela et al. 2018). Thus, while that adherence rate clearly contradicts those reported in the present review, it suggests that future studies that aim to investigate remotely administered, unsupervised digital exercise interventions need to be carefully planned and developed to promote adherence. Indeed, digital health applications can both incorporate adequate behavior-change approaches to increase user motivation as well as provide tailored interventional strategies and techniques, for instance goal-setting, to help increase engagement with the intervention (Ng et al. 2012). To this end, using digital technology to promote motivation and behavior change through tailoring, the intervention to the individual user and moving beyond the one-size-fits-all strategy could potentially result in higher adherence and consequently larger effects, although this warrants further confirmation (Horner et al. 2017; Tate et al. 2015).

### Limitations and strengths

A limitation of the present review is that merely five studies of very different nature met the inclusion criteria and were reviewed. Given the large heterogeneity in study design and that two studies did not have a control group, it was not justified to perform a meta-analysis. Thus, additional high-quality randomized controlled trials with a more similar design are needed before firm conclusions can be drawn. Future trials should also employ longer interventions as this may result in larger effects (Clark 2016), which would be valuable in order to make inferences on sustained long-term benefits. Furthermore, none of the included studies objectively measured VAT. This would be valuable in order to explore the benefits of digital exercise on measures of central obesity more accurately, as WC alone may be an insufficient measure of central obesity given its inability to differentiate visceral from subcutaneous adiposity (Després et al. 2008). Objective measurements also facilitate the detection of small changes in obese individuals (Shuster et al. 2012) whereas anthropometric surrogates can be difficult to apply in these individuals with respect to their body shape, thus risking impaired precision in outcome assessment. Also, as reflected in our bias assessment, it was unclear whether all outcome assessments were blinded or not, and it is possible that this may have influenced the results of the WC measurements. Finally, additional studies would also enable future subgroup analyses based on key factors e.g., intervention specifics; duration; measurement tools; degree of obesity; and sex. Especially, considering that there appears to be sex-specific responses to exercise in terms of visceral and total fat loss (Kuk and Ross 2009; Link and Reue 2017).

The strengths of the present study include a systematic and well-conceptualized methodology, pre-registration of the study protocol and independent assessment of eligible studies and bias assessment. This systematic review is also timely in an era where the health care is becoming more digitalized (Kostkova 2015) and the prevalence of obesity and physical inactivity remains high. Furthermore, despite that a healthy diet is a well-established cornerstone in the treatment of obesity (Jensen et al. 2014), evaluating the unique effects pertaining to exercise is a strength and may have other advantages. There is no one-size-fits-all solution, and for some people, it may be easier to start an exercise regime than to change their dietary habits. By starting to exercise, one could also potentially experience positive spin-off effects, as shown in a recent study where 12 weeks of exercise decreased wanting scores for high-fat foods and trait binge eating in overweight and obese individuals (Beaulieu et al. 2020). Finally, in this review, we have highlighted the fact that while many digital health applications promoting physical activity exist on the market today, very few have been tested in controlled studies (Byambasuren et al. 2018). It is therefore of interest to not only develop novel and innovative digital solutions, but essentially, they need to be evaluated. Specifically, this review exposes a gap in the field of research on studies evaluating the effectiveness of digital exercise interventions for reducing central obesity both in the short and long term as no studies have not factored in the key indicator VAT into their design and development.

### Conclusions

In conclusion, this systematic review shows that evidence for the potential effects of digital exercise for reducing VAT is lacking, although digital exercise appears to be beneficial for reducing WC in the short term within at least overweight individuals. This review highlights the need for additional randomized controlled trials to confirm the findings with respect to WC, and further investigate the effects of digital exercise on VAT. Given the pandemic of physical inactivity and obesity, this area of research has high relevance with potentially important implications in the strive toward reducing the incidence of their associated complications and economic burden.

## Appendix

### Search strategy

(((((((clinical[Title/Abstract] AND trial[Title/Abstract]) OR clinical trials as topic[MeSH Terms] OR clinical trial[Publication Type] OR random*[Title/Abstract] OR random allocation[MeSH Terms] OR “intervention study” OR “experimental study” OR “experimental trial” OR (randomized controlled trial[Publication Type] OR (randomized[Title/Abstract] AND controlled[Title/Abstract] AND trial[Title/Abstract]))))) AND (((“telemedicine”[MeSH Terms] OR “telemedicine”[All Fields] OR “tele-medicine”[All Fields] OR “telehealth”[All Fields] OR “tele-health”[All Fields] OR “mhealth”[All Fields] OR “m-health”[All Fields] OR “mobile health”[All fields] OR “ehealth”[All Fields] OR “e-health”[All Fields] OR “smartphone”[MeSH Terms] OR “smartphone”[All Fields] OR “Cell Phone”[mesh] OR “cell phone”[All fields] OR “mobile phone”[All fields] OR “video games”[MeSH Terms] OR “information technology”[MeSH Terms] OR “videotape recording”[MeSH Terms] OR “videos”[All Fields] OR “video”[All Fields] OR “education, distance”[MeSH Terms] OR “distance education”[All Fields] OR (“online”[All Fields] AND “learning”[All Fields]) OR “online learning”[All Fields] OR “computer-assisted instruction”[MeSH Terms] OR (“computer-assisted”[All Fields] AND “instruction”[All Fields]) OR “computer assisted”[All Fields] OR “computerized”[All Fields] OR (“self”[All Fields] AND “instruction”[All Fields] AND “programs”[All Fields]) OR “virtual reality”[MeSH Terms] OR “internet”[MeSH] OR internet[All fields] OR “web based”[All fields] OR “web based”[All fields] OR “text messaging”[MeSH] OR “text messaging”[All fields] “text messages”[All fields] OR telecommunications[MeSH] OR telecommunications[All fields] OR electronic OR digital OR “video-based”[All fields] OR “video based”[All fields] OR “video instruction*” OR “video instruction*”[All fields] OR digitalized OR “audio instruction*”)))) AND (((“exercise”[MeSH Terms] OR “exercise”[All Fields] OR “exercises”[All Fields] OR (“physical”[All Fields] AND “activity”[All Fields]) OR “physical activity”[All Fields] OR (“physical”[All Fields] AND “activities”[All Fields]) OR “physical activities”[All Fields] OR (“physical”[All Fields] AND “exercises”[All Fields]) OR “physical exercises”[All Fields] OR (“aerobic”[All Fields] AND “exercises”[All Fields]) OR “aerobic exercises”[All Fields] OR (“isometric”[All Fields] AND “exercises”[All Fields]) OR “isometric exercises”[All Fields] OR “physical fitness”[MeSH Terms] OR (“physical”[All Fields] AND “fitness”[All Fields]) OR “physical fitness”[All Fields] OR “walking”[MeSH Terms] OR “walking”[All Fields] OR “running”[MeSH Terms] OR “running”[All Fields] OR “gymnastics”[MeSH Terms] OR “gymnastics”[All Fields] OR “resistance training”[MeSH Terms] OR “resistance training”[All Fields] OR “strength training”[All Fields] OR “weight lifting”[MeSH Terms] OR “weight lifting”[All Fields] OR “cycling”[All Fields] OR “yoga”[MeSH Terms] OR “yoga”[All Fields] OR “jogging”[MeSH Terms] OR “jogging”[All Fields])))) AND ((obesity, Abdominal[MeSH] OR “central obesity”[All fields] OR “abdominal fat”[MeSH Terms] OR (“abdominal”[All Fields] AND “fat”[All Fields]) OR “abdominal fat”[All Fields] OR “overweight” OR “intra-abdominal fat”[MeSH Terms] OR “obesity” OR (“intra-abdominal”[All Fields] AND “fat”[All Fields]) OR “intra-abdominal fat”[All Fields] OR (“visceral”[All Fields] AND “fat”[All Fields]) OR “visceral fat”[All Fields] OR “Waist circumference”[mesh] OR “waist circumference”[All fields] OR “waist hip ratio”[all fields] OR “sagittal abdominal diameter”[all fields]))) AND (((“adult”[MeSH Terms] OR “adult”[All Fields] OR “adults”[All Fields] OR “aged”[MeSH Terms] OR “elderly”[All Fields] OR “oldest”[All Fields] OR “older”[All Fields] OR “senior”[All Fields] OR “seniors”[All Fields]) AND (“obesity”[MeSH Terms] OR obese[all fields] OR “obesity”[All Fields] OR “bariatrics”[MeSH Terms] OR “bariatrics”[All Fields]) OR “weight loss”[MeSH Terms] OR (“weight”[All Fields] AND “loss”[All Fields]) OR “weight loss”[All Fields] OR (“weight”[All Fields] AND “reduction”[All Fields]) OR “weight reduction”[All Fields] OR “overweight”[MeSH Terms] OR “overweight”[All Fields])).
